# Kikuchi-Fujimoto disease in a tertiary care teaching hospital in Coastal South India: A 8-year retrospective study.

**DOI:** 10.12688/f1000research.109832.1

**Published:** 2022-05-04

**Authors:** Basavaprabhu Achappa, Nipuni Chamathka Herath, Bodhi Sebastian, Nikhil Victor Dsouza, PAVAN MANIBETTU RAGHURAM, Ramesh Holla, Nithyananda Chowta, Jyoti Ramanath Kini

**Affiliations:** 1 Department of Internal Medicine, Kasturba Medical College, Mangalore, Manipal Academy of Higher Education, Manipal, India; 2 Department of Community Medicine, Kasturba Medical College, Mangalore, Manipal Academy of Higher Education, Manipal, India; 3 Department of Pathology, Kasturba Medical College, Mangalore, Manipal Academy of Higher Education, Manipal, India

**Keywords:** Kikuchi-Fujimoto disease, Histiocytic necrotizing lymphadenitis, Lymphadenopathy, Fever, Kikuchi disease, Rare diseases.

## Abstract

**Background: **Kikuchi-Fujimoto disease (KFD) is a rare, benign condition of unknown etiology, presenting as cervical lymphadenitis. Lymphadenopathy is usually tender and maybe associated with systemic symptoms. Despite the extensive literature on this disease, it continues to be misdiagnosed owing to its misleading clinical presentation.

Methods:

A retrospective hospital-based descriptive cross-sectional study was conducted in tertiary care hospitals from 2011 to 2019. All patients with confirmed KFD diagnosis were included and after ethics committee approval the clinical details and histopathological data was retrieved from the medical records department and analyzed.

Results:

A total of 67 cases were included. The mean age was 26.9±11.3 years with a female: male ratio of 1.9:1. There were 50 patients with tender cervical lymphadenopathy which was the most common clinical presentation. The mean length and width of palpable lymph nodes were 2.3±1.0 cm and 2.2±0.7 cm respectively. Histology revealed proliferative stage in majority of patients ( *n*=40, 59.7%). Lymphadenopathy resolved in 83.6% within 2 months. There were 42 patients who had complete recovery with symptomatic treatment within a period of 9 months.

Conclusions:

KFD is prevalent in young, female patients of Asian descent and often presents as tender cervical lymphadenopathy. Early diagnosis with excisional lymph node biopsy is crucial to avoid unnecessary investigations and treatment. Treatment is symptomatic unless complicated, where steroid therapy is considered. KFD has an excellent prognosis with almost no risk of fatality.

## Introduction

Kikuchi-Fujimoto disease (KFD), also known as “histiocytic necrotizing lymphadenitis” is a rare condition of unknown etiology. This benign condition presents as cervical lymphadenopathy, usually tender and is often associated with systemic symptoms like fever. ^1^ Identified first by two Japanese pathologists, independent of each other in the year of 1972, KFD was noted to have a higher incidence in Asian patients ^2^ and continues to be misdiagnosed till date. ^1^ Therefore, the awareness of this condition amongst clinicians and pathologists alike would be fruitful as it would aid in the early detection and prevention of life-threatening sequelae such as malignancies. ^3^

KFD is seen more frequently in young adults, with a mean age between 20–30 years, ^4^ but it can occur in any age group. ^1^ Even though a female predominance is reported in many previous cases, some studies done in Asia show a male to female ratio of 1:1. ^1^ The rare involvement of the heart, liver and lungs increases the fatality of an otherwise self-limiting disease. ^2^

The most common clinical feature is cervical lymphadenopathy, with or without systemic features such as fever, fatigue, headache and night sweats. ^5^ The lymphadenopathy is usually under 4 cms, ^6^ and in most cases can be tender or painful. ^2^ Hepatosplenomegaly has been reported in a few cases. ^1^

Both the etiology and pathogenesis of this disease is still unknown. ^1^ Clinically and histologically, the disease has overlapping features with tuberculosis, lymphoma or systemic lupus erythematosus (SLE) ^4^ resulting in dilemmas in diagnosis and management. KFD can be seen in patients with previous history of SLE, it can coexist with SLE or complicate into SLE. ^7^ This association with SLE was more common in Asian than European patients. ^6^ Meanwhile, a significant number of patients were found to have underlying viral infections. ^6^

The gold standard investigation for diagnosis is by histopathological examination of an excisional biopsy obtained from the affected lymph node. ^7^ Histologically, altered lymph node architecture by nodules of necrosis in the cortex, paracortical expansion, apoptotic cells with accumulation of crescentic histiocytes, and the absence of granulocytes especially neutrophils are typical fine-needle aspiration cytology features seen in KFD. ^6^^,^^8^ Due to challenges faced in diagnosing KFD, three evolving histological patterns have been proposed by pathologists based on key morphologic features. They are proliferative, necrotizing and xanthomatous based on the dominant histological pattern. ^7^ Most patients have normal laboratory findings. ^1^ However, a few cases with mild anemia, elevated Erythrocyte Sedimentation Rate (ESR) and C-reactive protein (CRP) along with leucopenia and elevated Lactate Dehydrogenase (LDH) have been reported rarely. ^3^

Even though, this disease almost always runs a benign course and resolves in several weeks to months in most patients, ^6^^,^^9^ it is seen to increase the risk of SLE and lymphoma. ^7^ Treatment if necessary is symptomatic with analgesics and antipyretics. ^1^ KFD has a very low recurrence rate and only a few fatalities have been reported. ^1^ The use of corticosteroid therapy is still under debate for the management of recurrent cases. ^1^

Despite the extensive studies done on KFD, it continues to be misdiagnosed till date. The objective of this study was to promote awareness among clinicians and pathologists alike. Therefore, this study discusses the clinico-epidemiological presentations of the disease, the histomorphology commonly found in these cases and the importance of follow-up of these patients to ensure complete recovery and avoid possible sequelae and complications.

## Methods

The study was a retrospective hospital based descriptive cross-sectional study carried out among all patients diagnosed with KFD in Mangalore. The diagnosis was confirmed following histopathological examination of the excision biopsy of the lymph nodes of suspected patients in the Pathology Department in Kasturba Medical College (KMC), Mangalore. The inclusion criteria consisted of all the patients diagnosed with KFD in KMC teaching hospitals from 2011–2019, while exclusion criteria were patients not diagnosed with KFD and cases diagnosed before 2011 and after 2019.

The Institutional Ethics Committee (IEC) of Kasturba Medical College, Mangalore (Manipal Academy of Higher Education) approval (Reference No. IEC KMC MLR 03-17/41) was obtained. The permission to access the medical records of KFD patients and a waiver of consent was obtained from the Institutional ethics committee.

The records of KFD patients during the period of April 2011 to April 2019 were reviewed and information on demographics, clinical profile and histopathological data of KFD patients seeking medical care was recorded in a data extraction sheet. The proforma included the relevant clinico-epidemiological information about the patient inclusive of clinical presentation, associated co-morbidities, examination findings, investigations done, pathological morphology as well as any complications or sequelae on follow-up over a period of 9 months.

Histopathological examination of the excision biopsy of all suspected cases of KFD was carried on formalin-fixed paraffin embedded sections stained with Hematoxylin and Eosin, Ziehl–Neelsen stain, Periodic acid-Schiff and Gomori Methenamine silver stains in the Pathology Department. The results obtained were categorized into 3 phases as proliferative phase, xanthomatous phase and necrotizing phase. The classic presentation of the proliferative stage was histiocytes, dendritic cells, lymphocytes and nuclear fragments. In the xanthomatous phase the predominant feature was foamy histiocytes within the lesions. Necrotizing phase typically showed extensive necrosis with karyorrhectic nuclear debris and coagulative necrosis and complete loss of lymph node architecture. The diagnosis of KFD was confirmed based on these morphological findings.

Data collected was analyzed using IBM Corp. Released 2017. IBM SPSS Statistics (RRID:SCR_016479) for Windows, Version 25.0. Armonk, NY: IBM Corp to analyze the clinico-epidemiological data, clinical presentation, local examination findings, histopathological presentation as well as outcomes on follow-up. This data was expressed as proportions, mean, standard deviation, median and Inter Quartile Range (IQR).

## Results

The baseline characteristics of the patients are shown below in Table 1. The age of our study population ranged from 4 to 77 years. The mean age was 26.9±11.3 years and a median age of 27 years (IQR: 18–32 years). As shown below KFD commonly affects younger adults between the ages of 21 to 40 years ( *n*=44, 65.7%) and was seen more in female patients ( *n*=44, 65.7%). When the occupation of the study population was analyzed, most of the patients were found to be homemakers ( *n*=27, 40.3%).

**Table 1.** Baseline characteristics of Kikuchi Fujimoto disease patients (n=67).

Baseline Characteristics	Number (n)	Percentage (%)
**Age group (years)**		
<21	18	26.9
21-40	44	65.7
41-60	03	04.5
61-80	02	03.0
**Sex**		
Female	44	65.7
Male	23	34.3
**Occupation**		
Homemaker	27	40.3
Minors/students	21	31.3
Business	14	20.9
Semi-skilled worker	02	03.0
Retired	01	01.5

Table 2 demonstrates the distribution of chief complaints at presentation in KFD patients. A vast majority of the patients presented with tender swelling over the side of the neck ( *n*=50, 74.6%). Following local examination, excision biopsies were performed on patients with positive findings. These biopsies revealed, the length and width of the cervical lymph nodes ranged from 0.5 to 5 cm and 1 to 5 cm respectively. The excised cervical lymph nodes had a mean length of 2.3±1.0 cm and mean width of 2.2±0.7 cm.

**Table 2.** Distribution pattern of the clinical presentation of KFD (n=67).

Presenting complaints	N *	Percentage (%)
Tender swelling over side of neck	50	74.6
Fever	35	52.2
Hepatosplenomegaly	10	14.9
Non-tender neck swelling	07	10.4
Axillary lymph node enlargement	06	09.0
Associated cough	03	04.5

* Multiple responses.

Patients also presented with complaints of fever ( *n*=35, 52.2%) and hepatosplenomegaly ( *n*=10, 14.9%) due to the systemic involvement of the disease. Hepatosplenomegaly was assessed by abdominal examination in all the patients. Computerized tomography (CT) scan of the abdomen was used for the patients with positive findings on clinical examination. Axillary lymph nodes were assessed by clinical examination and were found to be enlarged in 6 patients.

The histopathological profile of all patients was analyzed after classifying the findings of excision biopsies into the three morphological stages ( Table 3). Most of the patients presented during the proliferative stage ( *n*=40, 59.7%). The duration of lymphadenopathy in KFD patients ranged from a few days to 6 months ( Table 4). In most patients, lymphadenopathy resolved in less than 2 months ( *n*=56, 83.6%).

**Table 3.** Histopathological profile of Kikuchi Fujimoto disease patients (n=67).

Histopathological profile of KFD	n	Percentage (%)
Proliferative phase	40	59.7
Necrotizing phase	16	23.9
Xanthomatous phase	11	16.4

**Table 4.** Duration of lymphadenopathy of KFD patients (n=67).

Duration of lymphadenopathy	n	Percentage (%)
0 to15 days	24	35.8
15 to 2 months	32	47.8
2 months to 6 months	09	13.4
>6 months	02	03.0

The patients were followed-up over a period of 9 months to observe the outcomes of KFD. A vast majority of the patients recovered with symptomatic treatment ( *n*=42, 62.7%). Here, the lymphadenopathy was assessed using ultrasound examination of neck once the symptoms improved following treatment. Meanwhile steroid use was successful in treatment of KFD patients with systemic symptoms ( *n*=17, 25.4%), and one such patient presented with signs of neurological involvement which also resolved following treatment with steroids. A few patients were found to have a self-limiting course of KFD ( *n*=6, 12.2%). Recurrence of KFD was observed after treatment in 2 patients in this study. Both these patients developed tuberculosis over the next 2 to 3 months. Antinuclear antibody (ANA) levels were elevated in 3 patients, one of these patients was later diagnosed with SLE (positive for dsDNA). One elderly male patient who was diagnosed with necrotizing lymphadenitis of axillary nodes developed generalized lymphadenopathy, a repeat fine needle aspiration cytology (FNAC) and excision biopsy 6 months later confirmed a diagnosis of Non-Hodgkin Lymphoma.

## Discussion

KFD is a rare, self-limiting condition that is usually prevalent in Asian countries, clinically presenting as tender cervical lymphadenopathy with or without systemic symptoms. The etiology of the disease is yet to be uncovered, although certain studies have suggested an underlying viral infection or autoimmune disease to trigger the onset of KFD. ^2^ A retrospective hospital-based descriptive cross-sectional study was conducted on 67 patients diagnosed with KFD, to determine the sociodemographic profile, clinical presentation, and the outcome of the disease.

The mean age of KFD prevalence was found to be 27.1 years in this study while in a study done in Sub-Saharan Africa, an average of 21 years was recorded. ^10^ The youngest patient in our study was a 4-year-old girl and the eldest, a 77-year-old man. Of the study population, 16 were children (ranging from 4–18 years old) with confirmed cases of KFD. A study done on pediatric cases of KFD by Guleria S *et al.* reported a mean age of 10.8 years. ^11^ A study based on the characteristics of KFD based on age showed the mean age of children and adults to be 13.2 ± 4.8 and 32.7 ± 8.8 years respectively, which was similar to the range observed in our study. ^12^

On analysis of the gender prevalence, a female predominance of 1.91:1 was observed which was similar to a study done in South India which had a significant female majority of 2.4:1. ^9^ A ratio of 1:1 was observed in the results obtained from a study conducted by Pepe F *et al.* ^5^ in Italy. A reversal of the gender ratio was observed in two studies carried out in Korea and North India with a significant male majority of 2.8:1 ^13^ and 2:1 ^11^ respectively.

Younger females were noted to be more predisposed to KFD in our study with ages ranging from 4 to 38 years with all considerably younger than their male counterparts. A case series of 9 patients was conducted by Abeysekara RA *et al.*, where all the cases were female patients in the age group of 12–30 years. ^14^ Adhikari RC *et al.* carried out a similar study where 5 out of the 6 cases were females and the age range were 13–32 years. ^15^

The most common clinical presentation of these patients was tender swelling on the side of the neck (74.6%). Similar results were obtained from a study done in Michigan with 60–90% of the cases having posterior cervical lymphadenopathy. ^1^ Systemic symptoms such as fever (52.2% of the cases) and hepatosplenomegaly (14.9% of the cases) was found abundantly in this study. Fever was noted to be associated frequently with tender cervical lymphadenopathy ( *n*=30, 44.8%). This coexistence of symptoms was replicated in the Michigan study in 35–77% of the patients. ^1^ Tender cervical lymphadenopathy was the most common symptom in studies done in Italy ^5^ (60–98%), Saudi Arabia ^2^ (56–98%) and Michigan ^1^ (60–90%). The involvement of axillary lymph nodes was encountered in 9% of the cases in this study, whereas 13% were reported in a study conducted by Supari D *et al*. ^9^

One 22-year-old male patient in our study presented with symptoms of cervical lymphadenopathy for 15 days duration associated with systemic symptoms of fever, neck stiffness and meningitis. He was treated with steroids and tapered over a period of one month. Follow-up over 2 years revealed that lymphadenopathy subsided in 3 months and the patient recovered fully with no recurrence reported. In correlation to the above case, a study done in Japan reported 5 cases of recurrent KFD associated aseptic meningitis which resolved within several months with 3 of the cases requiring steroids. ^16^ Thus, the use of steroids in patients with recurrence of KFD with neurological involvement is considered beneficial following extensive investigation to ensure its safety. ^16^^,^^17^

The histopathology findings of the excisional lymph node biopsy remains a gold standard for confirming KFD. In this study, the findings were grouped into 3 morphological phases as done in previous studies ^5^^,^^7^^,^^8^ and a vast majority of the confirmed cases presented in proliferative phase of KFD ( *n=*40, 59.7%). Two of the patients whose lymph nodes showed proliferative phase with histiocytic aggregates later developed tuberculosis. Thus, histology of early tuberculosis can mimic KFD resulting in misdiagnosis. Similarly, two patients with predominance of necrosis went on to be diagnosed with SLE and Non-Hodgkin’s Lymphoma. Follow-up is thus crucial in KFD patients. Immunohistochemistry studies are ancillary techniques to support or exclude a diagnosis of lymphoma in suspicious cases.

A study conducted in Bangalore revealed that the duration of lymphadenopathy lasted from 1 week to 3 months, ^9^ however our study results suggested a longer duration of symptoms and it varied from 2 weeks to 6 months. The follow-up outcomes our patients over a period of 9 months revealed that all the cases completely recovered with no complications.

Regarding the treatment aspect, majority of our patients recovered after symptomatic management (62.7%), which was the choice of initial treatment in previous studies as well. ^1^^,^^11^^,^^16^ One fourth of the patients in this study were treated successfully with steroids (25.4%) and a similar result was obtained in two more studies where 2 out of the 6 patients required steroid therapy ^11^ and the utilization of methylprednisolone exhibited a drastic response within 24 hours. ^6^

Studies conducted in Florida had self-limiting KFD which typically lasted 1 to 4 months ^6^ and in England their symptoms resolved spontaneously within 6 months. ^7^ On the contrary, even though KFD is known to be a self-limiting condition, our study showed that only a small fraction of patients recovered completely without any treatment (9%).

Recurrence is always a matter of concern for the treating physician. Studies done have reported a recurrence rate of 3 – 4 % ^1^^,^^2^^,^^18^ in adults which was similar to our study recurrence rate of 3%. In comparison to children, we observed no recurrence which was a paradoxical finding when compared to a study by Han HJ *et al.* ^16^ which had a recurrence of 27% after a one year follow-up. Recurrence can present as late as 8 years after initial presentation according to Deaver D *et al*. ^6^ Therefore, long-term follow-up is essential to determine the rate of recurrence of KFD.

## Conclusion

KFD is a rare, idiopathic condition which presents as a diagnostic challenge to both pathologists and clinicians due to its misleading presentation. Demographically, the disease is prevalent in young, Asian, female patients and often presents as cervical lymphadenopathy. Early diagnosis with excisional lymph node biopsy is crucial to avoid unnecessary investigations and treatment for this self-limiting condition. Treatment is only symptomatic unless complicated, where steroid therapy is considered. KFD has an excellent prognosis with almost no risk of fatality. Long term follow-up of patients is vital to look for recurrence, complications of this condition and a few of them can evolve into SLE, lymphoma and TB.

## Data availability

### Underlying data

Figshare: Underlying data for ‘Kikuchi-Fujimoto disease in a tertiary care teaching hospital in Coastal South India: A 8-year retrospective study. https://figshare.com/s/b11d971a3f430af2f3d4. CC BY 4.0 license.

This project contains the following underlying data:

- Data sheet in excel format

## Author contributions

**Basavaprabhu Achappa** - Conceptualization; Data curation; Formal analysis; Methodology; Project administration; Resources; Supervision; Writing - original draft, review & editing.

**Nipuni Chamathka Herath** - Data curation; Formal analysis; Resources; Writing - original draft, review & editing.

**Bodhi Sebastian** - Data curation; Formal analysis; Writing - original draft.

**Nikhil Victor Dsouza** - Data curation; Formal analysis; Resources; Supervision; Writing - original draft, review & editing.

**Pavan Manibettu Raghuram** - Conceptualization; Formal analysis; Methodology; Project administration; Supervision; Writing - original draft, review & editing.

**Ramesh Holla** - Formal analysis; Methodology; Project administration; Resources; Supervision; Writing - original draft, review & editing.

**Nithyananda Chowta** - Formal analysis; Project administration; Resources; Supervision; Writing - original draft, review & editing.

**Jyoti Ramanath Kini** - Data curation; Formal analysis; Resources; Supervision; Writing - original draft, review & editing.
