# Supplementary material for: Kikuchi-Fujimoto disease in a tertiary care teaching hospital in Coastal South India: A 8-year retrospective study
Source: F1000Res. 2022 May 4;11:492. [Version 1] doi: 10.12688/f1000research.109832.1 (PMC9226704; doi:10.12688/f1000research.109832.1)
Supplement: Peer review history

# Peer review: Reviewer response for version 1

**DOI:** 10.5256/f1000research.121383.r139410

**Published:** 2022-06-28

**Authors:** Farhan Fazal

**Affiliations:** 1 Al Rahba Hospital, Abu Dhabi, United Arab Emirates

This is a retrospective study of a very important misdiagnosed entity. The authors have elaborated on the clinical as well as histological features of the disease. Since the study was retrospective it is not clear how the follow-up was done?. The authors have commented on the duration of lymph nodes that the patients had, but did not mention if the patients came for regular follow-up or was it done whenever the patient came for follow-up. Also what type of steroids and their dosage are used have not been mentioned. Overall the paper is well written.

Is the work clearly and accurately presented and does it cite the current literature?

Yes

If applicable, is the statistical analysis and its interpretation appropriate?

I cannot comment. A qualified statistician is required.

Are all the source data underlying the results available to ensure full reproducibility?

Partly

Is the study design appropriate and is the work technically sound?

Yes

Are the conclusions drawn adequately supported by the results?

Yes

Are sufficient details of methods and analysis provided to allow replication by others?

Yes

Reviewer Expertise:

Infectious disease

I confirm that I have read this submission and believe that I have an appropriate level of expertise to confirm that it is of an acceptable scientific standard.

# Peer review: Reviewer response for version 1

**DOI:** 10.5256/f1000research.121383.r139408

**Published:** 2022-06-23

**Authors:** Chaitanya Tellapragada Achyutha Krishna

**Affiliations:** 1 Division of Clinical Microbiology, Department of Laboratory Medicine, Karolinska Institute, Stockholm, Sweden

The article by Achappa B *et al.* is well written and provides important information regarding the baseline demographic and clinical features of patients with Kikuchi-Fujimoto disease (KFD) in South India. 

 I have few minor suggestions for the authors to add in their discussion:

- How many of these 67 patients were subjected to microbiological investigations for infectious aetiology (mainly viral infections) at the time of their initial presentation.
- Histopathological profiles of the study subjects are described in table 3 and duration of lymphadenopathy in table 4. It would be interesting if the authors could further discuss (based on their experience or previous studies) whether patients with any one or more of the three morphological phases (by histopathology or based on duration of lymphadenopathy) would benefit more from the steroid therapy.

Is the work clearly and accurately presented and does it cite the current literature?

Yes

If applicable, is the statistical analysis and its interpretation appropriate?

Not applicable

Are all the source data underlying the results available to ensure full reproducibility?

No source data required

Is the study design appropriate and is the work technically sound?

Yes

Are the conclusions drawn adequately supported by the results?

Yes

Are sufficient details of methods and analysis provided to allow replication by others?

Yes

Reviewer Expertise:

Microbiology; Epidemiology.

I confirm that I have read this submission and believe that I have an appropriate level of expertise to confirm that it is of an acceptable scientific standard.

# Peer review: Reviewer response for version 1

**DOI:** 10.5256/f1000research.121383.r139409

**Published:** 2022-06-13

**Authors:** Thejaswi K. Poonacha

**Affiliations:** 1 Department of Internal Medicine, University of Minnesota Medical Center, Minneapolis, USA

The authors have done an excellent retrospective observational study on Kikuchi-Fujimoto disease (KFD). While the sample size is relatively small, and no conclusions of real statistical significance can be concluded, it is still a landmark article considering the 8 year study, and the similarities noted in other studies. The authors' conclusion cannot be overstated with regard to the need for follow up in these patients. Nevertheless, this study deserves full merit.

 Please consider the following points:

1. The authors particularly focus on the clinical signs and symptoms, histopathology and the demographics. For the sake of completeness, they should include pathophysiology of the disease. ^1^
2. The authors could elaborate on the viral etiology of disease for academic significance. ^2^
3. As a follow-up project, I urge the authors to consider doing a meta-analysis study for this disease. There has been a meta analysis done on the causative agents.

Is the work clearly and accurately presented and does it cite the current literature?

Yes

If applicable, is the statistical analysis and its interpretation appropriate?

Yes

Are all the source data underlying the results available to ensure full reproducibility?

Yes

Is the study design appropriate and is the work technically sound?

Yes

Are the conclusions drawn adequately supported by the results?

Yes

Are sufficient details of methods and analysis provided to allow replication by others?

Yes

Reviewer Expertise:

Hematology/ Oncology

I confirm that I have read this submission and believe that I have an appropriate level of expertise to confirm that it is of an acceptable scientific standard.

## References

[1] : Apoptotic cell death in Kikuchi's disease: a TEM study. *Acta Otolaryngol Suppl* .1998;538:250-3 9879430

[2] : Causative agents of Kikuchi-Fujimoto disease (histiocytic necrotizing lymphadenitis): a meta-analysis. *Int J Pediatr Otorhinolaryngol* .2014;78(11) : 10.1016/j.ijporl.2014.08.019 1890-7 10.1016/j.ijporl.2014.08.019 25200851
